# In patients undergoing oesophagectomy does postoperative home enteral nutrition have any impact on nutritional status?

**DOI:** 10.1093/icvts/ivac120

**Published:** 2022-05-05

**Authors:** Xiaokun Li, Jianrong Hu, Jianfeng Zhou, Pinhao Fang, Yong Yuan

**Affiliations:** 1 Department of Thoracic Surgery, West China Hospital, Sichuan University, Chengdu, China; 2 Anesthesia Operation Center of West China Hospital/West China School of Nursing, Sichuan University, Chengdu, China

**Keywords:** Review, Home enteral nutrition, Oesophagectomy, Nutritional status

## Abstract

A best evidence topic in thoracic surgery was written according to a structured protocol. The question addressed was ‘In patients undergoing oesophagectomy does postoperative home enteral nutrition have any impact on nutritional status?’ Altogether, 50 articles were found using the reported search, of which 5 studies represented the best evidence to answer the clinical question. This consisted of 1 systematic review including a meta-analysis of 9 randomized controlled trials (RCTs), 3 RCTs and 1 cohort study. Main outcomes included loss of body weight and body mass index (BMI), change of serum albumin, haemoglobin, total protein and prealbumin, rates of nutritional risk patients and score value of patient-generated subjective global assessment. The meta-analysis concluded that there were significant differences in the loss of body weight and BMI between 2 groups, with higher values observed in the HEN group than that in the control group. One RCT showed that patients receiving HEN had a significantly lower weight loss compared with the control group. However, in another RCT, there was no significant difference between 2 groups in the loss of weight and body BMI. The available evidence shows that patients receiving home enteral nutrition yielded a significantly better BMI and lower decrease in body weight than those without after surgical resection of oesophageal cancer. We conclude that HEN could serve as an effective intervention for patients undergoing oesophagectomy. Moreover, the optimal time for patients receiving HEN could be 4–8 weeks after discharge. Feeding via jejunostomy and nasointestinal tube are feasible and safety approaches for HEN.

## INTRODUCTION

A best evidence topic was constructed according to a structured protocol. This is fully described in the ICVTS [1].

## THREE-PART QUESTION

In [patients undergoing oesophagectomy] does [postoperative home enteral nutrition] have any impact on [nutritional status] including body weight and serum nutrition-related indicators?

## CLINICAL SCENARIO

A patient newly diagnosed with oesophageal squamous cell carcinoma has undergone oesophagectomy with lymphadenectomy in our clinic. The patient asked whether he needs extra nutrition in addition to oral diet after discharge from hospital. One of my colleagues answered that there was evidence that home enteral nutrition was associated with better clinical outcomes of patients undergoing oesophagectomy after discharge from hospital. You resolve to check the literature yourself.

## SEARCH STRATEGY

We searched Medline by using the PubMed interface from 1950 to September 2018 with the following search terms: (oesophageal cancer[Title/Abstract] OR oesophageal carcinoma[Title/Abstract] OR oesophageal tumour[Title/Abstract] OR oesophageal neoplasm[Title/Abstract] OR oesophageal cancer[Title/Abstract] OR oesophageal carcinoma[Title/Abstract] OR oesophageal tumour[Title/Abstract] OR oesophageal neoplasm[Title/Abstract] OR oesophagectomy[Title/Abstract] OR oesophagectomy [Title/Abstract]) AND (home enteral nutrition[Title/Abstract] OR home enteral feeding[Title/Abstract] OR home oral feeding[Title/Abstract] OR home oral nutrition[Title/Abstract]).

## SEARCH OUTCOME

A total of 50 articles were found using the reported search from Medline. From these, 5 articles were identified that provided the best evidence to answer the question. They are presented in Table 1.

**Table 1:** Best evidence papers

Author, date, journal and country	Patient group	Outcomes	Key results	Comments
Study type (level of evidence)				
Liu *et al.* (2020), Medicine, China [2] Systematic review with meta-analysis of RCTs (level 1a)	Meta-analysis of 9 RCTs included 757 participants (HEN versus control: 376 vs 381); the sample size of each study ranged from 25 to 75; patients’ pathological stage was stage I to III; the patients were aged between 53 and 67.4; and the intervention time ranged from 1 to 4 months.	Loss of body weight Loss of BMI Change of serum albumin Change of haemoglobin Change of total protein Change of prealbumin Rates of nutritional risk patients Score value of PG-SGA	HEN (*n* = 170) versus control (*n* = 170): WMD 3 kg, 95% CI 2.36–3.63, *P* < 0.001; HEN (*n* = 216) versus control (*n* = 221): WMD 0.97 kg/m^2^, 95% CI 0.74–1.21, *P* < 0.001 HEN (*n* = 249) versus control (*n* = 252): WMD 3.43 g/l, 95% CI 2.35–4.52, *P* < 0.001 HEN (*n* = 139) versus control (*n* = 147): WMD 7.23 g/l, 95% CI 5.87–8.59, *P* < 0.001 HEN (*n* = 198) versus control (*n* = 195): WMD 5.13, 95% CI 3.7–6.56, *P* < 0.001 HEN (*n* = 125) versus control (*n* = 122): WMD 23.58 mg/l, 95% CI 0.05–47.11, *P* = 0.05 HEN (*n* = 35) versus control (*n* = 53): RR = 0.64, 95% CI 0.48–0.84, *P* = 0.001 HEN (*n* = 91) versus control (*n* = 99): WMD 2.17, 95% CI 2.6–1.74, *P* < 0.001	The language of included studies was limited to English and Chinese The study did not use subgroup analysis to stratify various patients The time and approach of home enteral nutrition in different studies had heterogeneity The time of detection of nutritional status in different studies was various
Bowrey *et al.* (2015), Trials, UK [3] Open-label RCT (level 1b)	41 patients underwent oesophagectomy or total gastrectomy for cancer. Twenty received 6 weeks of HEN, and 21 received standard care. HEN was offered via jejunostomy tubes.	Loss of weight (kg) (6 weeks after hospital discharge) Loss of weight (%) (6 weeks after hospital discharge)	HEN group: −3.8 ± 3.5 kg Control group: −8.6 ± 3.9 kg HEN group: −4.6 ± 3.9 % Control group: −9.7 ± 4.8 %	Open-label RCT with small-sample size The randomization process is not clear The study did not compare 2 groups with statistical analysis
Gavazzi *et al.* (2016), Eur J Cancer, Italy [4] Multicentre open-label RCT (level 1b)	79 patients with upper gastrointestinal cancer. Thirty-eight received HEN and 41 received standard care. HEN was offered via jejunostomy tubes.	Loss of weight (2 months after hospital discharge) Loss of weight (6 months after hospital discharge)	HEN group: −0.3 ± 3.9 kg; −0.5% compared with baseline Control group: −3.6 ± 4.8 kg; −5.8% compared with baseline (*P* = 0.0031) HEN group: −0.4 ± 5.6 kg; −0.6% compared with baseline Control group: −2.4 ± 5.0 kg; −3.8% compared with baseline	Multicentre, controlled, open-label, two-parallel groups, randomized clinical trial Small-sample size The trial was stopped since HEN showed increased efficacy over counselling only (2 months after hospital discharge)
Li *et al.* (2020), Nutrition, China [5] Single-blind RCT (level 1b)	62 patients underwent oesophagectomy. Thirty received HEN and 32 received SEN. HEN was offered via jejunostomy tubes.	Loss of weight (1 month after surgery) Loss of BMI (1 month after surgery)	HEN group: −2.08 ± 1.95 kg SEN group: −3.53 ± 1.79 kg (*P* = 0.13) HEN group: −1.01 ± 0.72 kg/m^2^ SEN group: −1.29 ± 0.69 kg/m^2^ (*P* = 0.12)	Parallel-group, randomized, single-blind clinical trial Small-sample size
Chen *et al.* (2021), Ann Palliat Med, China [6] Single-centre prospective non-randomized observational trial (level 2b)	60 patients underwent oesophagectomy. Thirty received HEN and 30 received conventional nutrition. HEN was offered via the nasointestinal tube.	BMI (8 weeks after surgery) Serum albumin (8 weeks after surgery)	HEN group: 19.1 ± 4.8 Control group: 16.1 ± 4.3 (*P* < 0.05) HEN group: 40.1 ± 5.9 Control group: 31 ± 3.8 (*P* < 0.05)	Single-centre retrospective non-randomized cohort study Small-sample size

BMI: body mass index; CI: confidence interval; HEN: home enteral nutrition; PG-SGA: patient-generated subjective global assessment; RCTs: randomized clinical trials; SEN: standard enteral nutrition; WMD: weighted mean difference.

## RESULT

Liu *et al.* [2] performed a meta-analysis of 9 randomized controlled trials (RCTs) including 757 participants [home enteral nutrition (HEN) versus control: 376 vs 381] to study the efficacy of home enteral nutrition in patients undergoing oesophagectomy. The main findings were that HEN improved nutrition status compared with an oral diet in oesophageal cancer patients after surgery, without increasing adverse reactions. The results favoured the use of HEN in oesophageal cancer patients after discharge. The loss of body weight and body mass index (BMI) was reported in 4 (*n* = 340) and 5 (*n* = 437) RCTs, respectively. The data of the meta-analysis showed that patients in the HEN group yielded significantly higher value of body weight and BMI after nutrition intervention between 2 groups [loss of body weight: weighted mean difference (WMD) 3 kg, 95% confidence interval (CI) 2.36–3.63, *P* < 0.001; loss of BMI: WMD 0.97 kg/m^2^, 95% CI 0.74–1.21, *P* < 0.001]. The change in serum albumin, haemoglobin, total protein and pre-albumin was reported in 6 (*n* = 501), 3 (*n* = 286), 5 (*n* = 393), and 3 (*n* = 247) RCTs, respectively. Meanwhile, the HEN group yielded significantly higher concentrations of serum albumin (WMD 3.43 g/l, 95% CI 2.35–4.52, *P* < 0.001), serum haemoglobin (WMD 7.23 g/l, 95% CI 5.87–8.59, *P* < 0.001) and serum total protein (WMD 5.13, 95% CI 3.7–6.56, *P* < 0.001). No significant differences were observed in serum prealbumin (WMD 23.58 mg/l, 95% CI 0.05–47.11, *P* = 0.05). Two RCTs including 145 participants reported the rates of nutritional risk patients at the end of their HEN; a significant advantage favouring the HEN group was observed (relative risk (RR) = 0.64, 95% CI 0.48–0.84, *P* = 0.001). Two studies reported the score value of patient-generated subjective global assessment; the patient-generated subjective global assessment scores of the HEN group were significantly lower than that of the control group (WMD 2.17, 95% CI 2.6–1.74, *P* < 0.001).

Bowrey *et al.* [3] conducted an RCT including 41 patients (20 received 6 weeks of HEN, 21 received standard care) to explore the impact of HEN on the nutrition status of patients undergoing oesophagectomy or total gastrectomy. Home enteral nutrition was administered via 6 weeks of home jejunostomy feeding. Eleven (50%) patients developed minor jejunostomy complications in the HEN group (*n* = 22). They used the loss of weight as the first outcome to assess the nutrition status of patients. The study concluded that the weight loss was significantly associated with the approach of nutrition support. Patients administered HEN had a significantly less weight loss (kg) (HEN group: −3.8 ± 3.5 kg vs standard care group: −8.6 ± 4.7 kg) compared with the patients in the control group. When comparing the weight loss calculated by percentage (%), patients in the HEN group still had a lower weight loss (HEN: −4.6 ± 3.9% vs standard care group: −9.7 ± 4.8%) compared with the control group. However, their study existed obvious shortcomings. First, the randomization process is not clear. Second, their study did not compare 2 groups with statistical analysis.

Gavazzi *et al.* [4] launched a multicentre open-label small-sample RCT including a total of 79 patients (38 received HEN, 41 received standard care) focusing on patients with upper gastrointestinal cancer. Adult (>18 years) patients with documented cancer of the upper gastrointestinal tract who were candidates for major elective surgery and presented a preoperative nutritional risk score ≥3 according to the NRS 2002 tool were eligible. No complications associated with HEN were reported. They employed body weight loss to evaluate the nutritional status of patients 2 months after hospital discharge. The results showed that patients who received HEN had a significantly lower weight loss compared with the control group (−0.3 ± 3.9 vs −3.6 ± 4.8 kg; *P* = 0.0031). However, this study could not perfectly present the advantages of HEN since patients with gastric and pancreatic cancer were also included in the study.

Recently, Li *et al.* [5] conducted a high-quality single-blind RCT including 62 patients undergoing oesophagectomy [30 received HEN, 32 received standard enteral nutrition (SEN)]. In their study, an enteral feeding pump was used to infuse enteral nutrition (Peptisorb, Nutricia) via jejunostomy tube postoperatively. After discharge, patients in the HEN group were instructed to independently administer jejunostomy feeds at home for 1 month while jejunostomy tubes of the control group were removed. In the HEN group, patients received normal food via oral intake (light diet at the first week, normal diet after the first week) combined with enteral nutrition (500 ml/day) via jejunostomy tube. Relatively, patients in the standard group only received normal food via oral intake. Two patients in the HEN group developed skin inflammation and had their jejunostomy tubes removed during 4 weeks. The loss of weight (kg), BMI (kg/m^2^), lean body mass (kg), skeletal muscle mass (kg), height-adjusted appendicular skeletal muscle mass (kg/m^2^) and body cell mass (kg) were compared between 2 groups 30 days after discharge. The study showed that HEN was associated neither with weight loss (HEN: −2.08 ± 1.95 vs SEN: −3.53 ± 1.79 kg; *P* = 0.13) nor with the loss of BMI (HEN: −1.01 ± 0.72 vs SEN: −1.29 ± 0.69 kg/m^2^; *P* = 0.12). Meanwhile, there were no significant differences were found between 2 groups in the loss of lean body mass (HEN: −2.12 ± 1.45 vs SEN: −2.79 ± 1.56; *P* = 0.086) and BMI (HEN: −2.35 ± 2.21 vs SEN: −3.22 ± 2.57; *P* = 0.16). However, the HEN group yielded significantly less loss of skeletal muscle mass (HEN: −2.22 ± 1.78 vs SEN: −3.15 ± 1.23 kg; *P* = 0.036) and height-adjusted appendicular skeletal muscle mass (HEN: −1.21 ± 0.41 vs SEN −1.43 ± 0.34 kg/m^2^; *P* = 0.025) compared with the SEN group. Their study also explored the 2-year survival outcome between 2 groups. Two-year progression-free survival and overall had no significant differences in survival curves comparing 2 groups (*P* = 0.36 and 0.29, respectively). The study concluded that 4 weeks of HEN is a safe and feasible nutritional strategy to improve nutritional status after oesophagectomy. Although there was no significant difference in survival between the HEN and SEN groups, HEN could still be more effective and beneficial to patients with defective nutritional status than SEN.

Most recently, Chen *et al.* [6] conducted a retrospective cohort study including 60 patients undergoing oesophageal surgery (30 received HEN and 30 received conventional nutrition). In their study, enteral nutrition (EnSure^®^, Abbott Laboratories, the Netherlands) was offered via the nasointestinal tube after the second postoperative day. The regular diet was offered to both groups after discharge. In the HEN group, patients continued to receive enteral nutrition (6 standard scoops of EnSure^®^ dissolved in 200–250 ml warm water with frequency no less than 6 times/day) daily with or between meals for 8 weeks after discharge. However, patients in the conventional nutrition group only received regular diets after discharge. The study found that patients in the HEN group were significantly associated with high nutritional status (HEN malnourished rate: 10% vs conventional nutrition malnourished rate 50%; *P* < 0.05) 8 weeks after discharge. Meanwhile, the HEN group yielded a significantly higher BMI compared with the conventional nutrition group (19.1 ± 4.8 vs 16.1 ± 4.3 kg/m^2^; *P* < 0.05). Moreover, the HEN group also yielded a significantly higher level of serum albumin (HEN: 40.1 ± 5.9 vs conventional nutrition: 31 ± 3.8 mg/dl; *P* < 0.05) compared with the conventional nutrition group. The study concluded that elderly patients who underwent oesophagectomy could benefit from HEN, which can improve their nutritional status. Meanwhile, to optimize its efficacy, a HEN should last no less than 8 weeks after discharge.

## CLINICAL BOTTOM LINE

The available evidence shows that patients receiving home enteral nutrition yielded a significantly better BMI and lower decrease of body weight than those without after surgical resection of oesophageal cancer. We conclude that HEN could serve as an effective intervention for patients undergoing oesophagectomy. Moreover, the optimal time for patients receiving HEN could be 4–8 weeks after discharge. Feeding via jejunostomy and nasointestinal tube are feasible and safety approaches for HEN.
